# Complete Genome Sequence of a Recombinant Porcine Epidemic Diarrhea Virus Strain from Eastern China

**DOI:** 10.1128/genomeA.00105-13

**Published:** 2013-04-18

**Authors:** Bin Li, Haofei Liu, Kongwang He, Rongli Guo, Yanxiu Ni, Luping Du, Libin Wen, Xuehan Zhang, Zhengyu Yu, Junming Zhou, Aihua Mao, Lixin Lv, Yiyi Hu, Yang Yu, Haodan Zhu, Xiaomin Wang

**Affiliations:** Institute of Veterinary Medicine, Jiangsu Academy of Agricultural Sciences, Key Laboratory of Veterinary Biological Engineering and Technology, Ministry of Agriculture, Nanjing, Jiangsu Province, China

## Abstract

A field porcine epidemic diarrhea virus (PEDV) strain, JS2008, was isolated from stool samples of a piglet with acute diarrhea on a vaccinated farm in eastern China. We sequenced and analyzed the complete genome of strain JS2008, which will help increase our understanding of the molecular characteristics of the epidemic PEDV in China.

## GENOME ANNOUNCEMENT

Porcine epidemic diarrhea virus (PEDV), a member of the family *Coronaviridae*, is an enveloped virus with a single-stranded, positive-sense RNA and causes porcine epidemic diarrhea, an enteric disease characterized by acute watery diarrhea, dehydration, vomiting, and high mortality in nursery piglets (1, 2). Outbreaks of PEDV infection have been documented mainly in European and Asian countries since the disease was first recognized in an acute outbreak of diarrhea in pigs (1).

In the autumn of 2010, severe diarrhea in piglets emerged in China, affecting more than 1,000,000 piglets (3). A PEDV variant has been identified as a main causative agent in the disease (4–7). In this study, a PEDV strain, JS2008, was detected and identified in stool samples from a piglet with severe diarrhea on a vaccinated farm in eastern China. This strain can be easily isolated in Vero cells. Thus, we sought to analyze the genome sequence of PEDV strain JS2008 and understand its molecular characteristics.

Twenty pairs of oligonucleotide primers to amplify regions of the JS2008 genome were designed from alignments of available PEDV genomes. The PCR products were purified and cloned into the pMD18-T vector (TaKaRa) and sequenced with an ABI3730XL genome sequencer. The terminal sequences were acquired by using a kit for rapid amplification of cDNA ends (RACE) (Clontech, Japan).

The complete genome sequence of JS2008 is 27,953 nucleotides (nt) in length, excluding the poly(A) tail. The genome showed 99.7% and 98.0% nucleotide sequence similarity to the attenuated DR13 and CV777 vaccine strains. It shared 97.3 to 97.5% sequence identity with the PEDV variant that has occurred in recent years in China. The organization of the genome of JS2008 is similar to that of other reported PEDV genomes, with the characteristic gene order 5′-ORF1a/1b-S-ORF3-E-M-N-3′ (2, 8, 9). The ORF1a/1b gene is 20,321 nt in length. The sizes of the other genes, S, ORF3, E, M, and N, are 4,149 nt, 276 nt, 231 nt, 681 nt, and 1,326 nt, respectively. Sequence analysis showed that there was a 49-nt deletion in the ORF3 gene, and this characteristic was similar to a characteristic of the attenuated DR13. Meanwhile, the E gene was 21 nt longer than that of the attenuated DR13 and had the same sequence as that of the PEDV variant. These findings may indicate that JS2008 is a recombinant of the PEDV vaccine strain and the variant.

Phylogenetic analysis of the complete genome showed that JS2008 has a close relationship to the attenuated DR13 vaccine strain and a distant relationship to the prevalent PEDV variant in China. The availability of the genome sequence of JS2008 will facilitate future investigations of the epidemiology and evolution of PEDV.

### Nucleotide sequence accession number. 

The genome sequence of PEDV strain JS2008 was deposited in GenBank under accession number KC109141.
